# Gut Microbiota-Polyphenol Interactions in Chicken: A Review

**DOI:** 10.3390/ani10081391

**Published:** 2020-08-11

**Authors:** Yasir Iqbal, Jeremy J. Cottrell, Hafiz A.R. Suleria, Frank R. Dunshea

**Affiliations:** 1 School of Agriculture and Food, Faculty of Veterinary and Agricultural Sciences, The University of Melbourne, Parkville, VIC 3010, Australia; yasir.iqbal@student.unimelb.edu.au (Y.I.); jcottrell@unimelb.edu.au (J.J.C.); hafiz.suleria@unimelb.edu.au (H.A.R.S.); 2 Faculty of Biological Sciences, The University of Leeds, Leeds LS2 9JT, UK

**Keywords:** chicken, gut, microbiota, polyphenols, bioavailability, metabolites, modulation

## Abstract

**Simple Summary:**

Gut microbiota play crucial roles in digestion of feed and absorption of nutrients in fast growing chickens. Gut microbiota affects feed conversion ratio, body weight gain, apparent metabolizable energy, residual feed intake, and time taken to attain the desired weight, which have direct influence on the health and productivity of chickens. A normal gut microbiota is therefore very important for optimum health. Factors like the environment, heat stress, and housing conditions can cause detrimental changes in the gut resulting in poor health of birds and decreased production performance. Polyphenols can be used to improve gut health due to their established health benefits and strong antioxidant potential. The interaction between polyphenols and the gut microbiota further generates active metabolites, which can modulate the composition of the chicken gut microbiota. Because of the specificities of the gut microbiota-polyphenols interactions, current knowledge in this area is presented.

**Abstract:**

The gastrointestinal tract of the chicken harbors very complex and diverse microbial communities including both beneficial and harmful bacteria. However, a dynamic balance is generally maintained in such a way that beneficial bacteria predominate over harmful ones. Environmental factors can negatively affect this balance, resulting in harmful effects on the gut, declining health, and productivity. This means modulating changes in the chicken gut microbiota is an effective strategy to improve gut health and productivity. One strategy is using modified diets to favor the growth of beneficial bacteria and a key candidate are polyphenols, which have strong antioxidant potential and established health benefits. The gut microbiota-polyphenol interactions are of vital importance in their effects on the gut microbiota modulation because it affects not only the composition of gut bacteria but also improves bioavailability of polyphenols through generation of more bioactive metabolites enhancing their health effects on morphology and composition of the gut microbiota. The object of this review is to improve the understanding of polyphenol interactions with the gut microbiota and highlights their potential role in modulation of the gut microbiota of chicken.

## 1. Introduction

The gastrointestinal tract of chickens is composed of the crop, gizzard, proventriculus, duodenum, jejunum, ileum, caeca, large intestine (colon) and cloaca [1] all of which contain complex microbial communities with varying populations. These densely populated microbial communities in the gut compartments are collectively called the gut microbiota and includes bacteria, fungi, archaea, protozoa, and viruses, with bacteria the most abundant among all these. *Clostridium*, *Lactobacillus*, *Eubacterium*, *Bacteroides*, *Escherichia coli*, *Prevotella*, *Selenomonas*, *Streptococcus*, *Megasphaera*, *Fusobacterium,* and *Bifidobacterium* are among the most common bacteria in the chicken gut. The number and type of microbial communities in each section of the gut varies depending upon nutrient flow from the diet, immune responses of the host and the substances produced by this complex microbial system in the gut [2]. 

Both beneficial bacteria (associated with improved productivity) and harmful bacteria (associated with poor health and diseases) inhabit the gut of healthy birds. However, a dynamic balance is maintained in such a manner that beneficial Gram-positive bacteria generally dominate, constituting more than 85% of the bacterial population [3]. Some pathogenic bacteria like *Clostridium* sp. in young birds and *E. coli*, *Campylobacter,* and *Salmonella* in healthy adult birds are also common among other bacteria in the chicken gut [4]. The maintenance of this population in the gut is responsible for the digestion and absorption of nutrients, the development of immunity and disease resistance. A deviation in the normal gut microbiota results in “Dysbiosis” that designates to qualitative or quantitative imbalances of the microbial populations from the normal proportions in the gut. Dysbiosis can cause detrimental changes in the gut affecting the digestion of ingested food, nutrients absorption and mucosal barrier function leading to translocation of infectious bacterial species, and inflammatory response [5]. This can affect feed conversion ratio, productivity, performance, and overall health of poultry birds [6]. A normal gut microbiota is, therefore, very important for optimal health and productivity.

Different factors like environmental conditions, genetic background, age, and stress affect the gut microbiota with the diet being the most influential factor in the development of the gut microbiota and its functionality [7]. The type of feed is crucial in driving the gut microbial ecology in chicken as the undigested and unabsorbed components of feed can serve as substrates for microbial growth in the gut [8,9]. Various feed additives have been used to modulate the gut microbiota of chicken. Previously, antibiotics were used as growth promoters, but consumer awareness about harmful effects of antibiotics coupled with increasing antimicrobial resistance has resulted in increased regulatory oversight and a focus by industry and research to explore alternatives [10]. Feed supplementation with phytochemicals especially polyphenols can serve to fulfill this need as polyphenols and their metabolites can modulate changes in bacterial diversity and population in chicken gut due to their already established health promoting effects [11].

The beneficial properties of polyphenols have been credited to the formation of their biologically active metabolites and their ability to modulate changes in gut microbial populations [11]. The bioavailability of polyphenols is crucial for their effects on gastrointestinal tract and other systems of the body like antioxidant system, cardiovascular, and immune stimulation [12]. The literature regarding bioavailability status of polyphenols reports a low bioavailability, particularly for the proanthocyanidins in chickens [13]. Due to their low absorption, about 90% of phenolic compounds enter the colon unaltered and interact with colonic bacteria. The gut microbiota-polyphenols interactions play vital roles in modulation of the gut microbiota and not only alter the bacterial composition of gut but also improve the bioavailability of polyphenols by metabolizing them into absorbable metabolites. The altered microbial composition and bacteria-derived metabolites of polyphenols affects the development of the gut and can improve health and productivity of chicken.

## 2. Chicken Productivity and Its Relationship with the Gut Microbiota 

Poultry performance may be predicted by recording parameters like feed conversion ratio (FCR), body weight gain (BWG), apparent metabolizable energy (AME), residual feed intake (RFI), and time taken to attain desired weight. Feed conversion ratio can be designated as ratio of ingested feed to gain in body weight and is the most widely used parameter for measuring growth performance. The gut microbiota composition can affect FCR as it plays crucial roles in digesting feed and nutrient absorption. The presence of *Campylobacter* has been negatively linked with productive performance due to increased FCR [14]. In studies with the cecum, differences among bacterial communities linked to FCR were observed. In most of the studies, *Lactobacilli* were reported to be linked to higher FCR and *Faecalibacterium* genera were associated with lower FCR. No microbial colonies have been reported to be linked with higher or lower FCR in the jejunum in a study [15]. In contrast, another study linked *Lactobacillus*, *Fructobacillus*, *Paralactobacillus* with high FCR and *Leptotrichia*, *Pediococcus*, *Rohdococcus*, *Escherichia coli* with low FCR in the jejunum [16].

In the crop, *Bacteroidetes*, *Euryarchaeota*, *Ruminococcus,* and *Faecalibacterium* bacteria have been reported to be associated with high productivity, whereas, *Actinobacteria*, *Bifidobacterium,* and *Lactobacillus* were found to be associated with low productivity in terms of BWG [17]. *Clostridium coccoides* were linked with higher BWG, whereas, *Enterobacteria*, *E. coli* and *Shigella* were linked with lower BWG [18]. Some bacterial taxa in the ileum have been identified to be linked with productive performance of chicken. *Lactobacillus* species have been usually correlated with higher performance and better health [19]. In contrast, two studies have reported *Lactobacillus* species in the ileum to be associated with low productivity [20]. 

Prominent variations in microbial communities were observed in the cecum, which harbors the higher diversity of bacterial communities where *Lactobacillus salivarius*, *Lactobacillus crispatus*, *Lactobacillus aviarius*, *Clostridium lactatifermentans*, some bacteria belonging to *Ruminococcaceae* family, *Akkermansia*, *Faecalibacterium,* and *Bacteroides vulgatus* were related to improved performance [21], whereas, *Escherichia* and *Shigella* strains were correlated with lower fat digestibility and performance in broiler chickens [18]. In some studies with fecal samples, *Lactobacillus* and *Bacteroides* were reported to be associated with productive performance [22]. Hence, the composition of the gut microbiota can affect the productive performance of the chicken (Table 1). However, the relationship between the gut microbiota composition and productive performance of chickens has not yet been fully elucidated. Some authors have reported that high feed efficiency is associated with less diverse gut microbiota in chicken [20], while others have linked higher bacterial communities’ complexity and richness with better feed efficiency [21].

## 3. Gut Microbiota-Polyphenols Interaction and Effects on Chicken Gut Microbiota

Gut microbiota-polyphenols interaction is a two-way process in which the gut microbes metabolize polyphenols into simpler metabolites whereas polyphenols affect the growth and population of gut microbes by interfering with their metabolic activities [23] (Table 2). The bioavailability of polyphenols is also increased due their transformation into absorbable metabolites [11]. Particularly, *Lactobacilli* bacteria are capable of metabolizing polyphenols producing energy for use by cells and simpler compounds that can interfere with metabolic activities of gut bacteria. *Lactobacillus acidophilus* can deglucosylate plant glycosides and produce aglycones [24] and some microbial taxa (*Lactobacillus delbrueckii* and *Eubacterium ramulus*) can use these aglycones as nutritional substrates [25]. Others (*B. ovatus*, *Veillonella* sp. and *Ruminococcus productus)* can further metabolize aglycones via ring opening, breakdown of lactone, decarboxylation, dehydroxylation, demethylation, reduction, and isomerization [26].

Enzymatic action of colonic bacteria breaks down the basic structures of unabsorbed polyphenols and increases their bioavailability by transforming them into low molecular weight metabolites of varying bioactive potentials [27,28]. These low molecular weight polyphenol metabolites can persist in the plasma and can exhibit improved health promoting effects. While the greatest numbers of bacterial species inhabit the colon, a few of them (*E. coli*, *Lactobacillus* sp., *Bifidobacterium* sp., *Bacteroides* sp., and *Eubacterium* sp.) are known for metabolisms of polyphenols with their metabolic pathways [29].

The microbial derived metabolites and primary polyphenolic compounds can affect microbial composition of the gut and signaling pathways [35]. The strong antioxidant potential of polyphenols provides protection to the epithelial wall of the colon and mucosa and shapes the gastrointestinal environment for the growth of microbial communities in the colon by exerting prebiotic effect and antimicrobial action. Polyphenols affect epigenetic changes in intestinal tissues and modulate intracellular receptors and signaling molecules [36]. These phenolic compounds also affect the intestinal detoxification systems and exert antioxidant action on pre-colon epithelial cells [36]. Polyphenols provide protection to epithelial cells and prevent inflammation in the intestines resulting in improved gut barrier function [37].

Polyphenols suppress pathogenic bacteria on one hand while supporting beneficial bacteria by acting as prebiotics on the other hand. The antimicrobial properties of polyphenols are of prime importance and prevent the formation of biofilms in the gut by suppressing the growth of harmful bacterial species [26]. Polyphenols like quercetin, hydroxytyrosol, resveratrol, and phenolic acids exhibit antimicrobial properties against various pathogenic bacteria including *Helicobacter pylori* and *Salmonella* and are known to inhibit various pathogenic microbes. Resveratrol can suppress the population of *Escherichia coli* and can ameliorate the impact of heat stress in broilers [38]. Whereas, quercetin can suppress *S. enteritidis* and can affect pro-inflammatory gene expression [39]. The antimicrobial action of these compounds is accomplished by various mechanisms that include direct inhibition of certain bacterial species, reduction of adhesion ability of pathogenic bacteria, or disruption of ionic fluxes at the cell membrane [40]. The concentration of phenolic compounds in feed, selectivity of their antimicrobial action, and bacterial resistance to these natural antimicrobials affect the susceptibility of bacteria [40]. Improved nutrient utilization and increased bird performance have been linked with polyphenols due to inhibition of harmful bacteria (*E. coli*, *Clostridium*) adhesion, thereby preventing gut infections [25].

Polyphenols possess prebiotic action and support growth of selective bacteria by acting as a source of nutrient supply [41,42]. Due to “prebiotic-like” effect, polyphenols can enhance multiplication of favorable microbes such as the *Lactobacilli* and *Bifidobacteria* populations [43]. Ingestion of different polyphenols is linked to varying ratios of gut bacteria, *Bacteroides* and *Firmicutes*, favoring the growth of *Bacteroides* that possess a higher number of glycan-degrading enzymes. Feed supplemented with polyphenol-rich grapes enhanced *Enterococcus* population while decreasing *Clostridium* levels in chickens [25]. Feeding cranberry extract containing anthocyanins, flavanols, flavonols, and phenolic acids markedly reduced *Enterococcus* species population in broilers [44]. Polyphenols rich green tea and mulberry leaves are among the most widely-used plant components in poultry production [45] and are reported for selective inhibition of some disease-causing bacterial strains without impacting commensal bacterial communities. Sugarcane-derived polyphenol mix (Polygain) can improve meat stability in chicken [46]. Grape polyphenol extract can also improve oxidative stability of meat by favoring the growth of beneficial bacterial communities (*Lactobaccilli* and *Bifidobacteria*) in intestines [47]. Addition of polyphenols-rich powdered mulberry leaves in feed of chicken resulted in increased population of *Prevotella*, *Megamonas,* and *Bacteroides* in the gut of chicken. These reports are indicative of the fact that powdered mulberry leaves and green tea (rich in polyphenols) possess potential to influence gut microbiota of chicken [48].

Polyphenol rich blueberry byproducts also favored population of certain bacterial species including *Bacteroidetes* in ceca and cloaca of birds [49], whereas cranberry pomace fractions caused inhibition of AMR *Salmonella serovars* by interfering with cell metabolic activities, nutrient utilization, and virulence capacity of *Salmonella enteritidis* in broilers [50]. Berry pomaces suppress *C. perfringens* pathogenesis in broilers and promotes gut health [51]. Wine polyphenols can promote *Bifidobacteria* and *Lactobacilli* growth, while inhibiting *Clostridia* [25].

Morphological traits (length of villi, crypt depth, and height) are indicators of gut health. Lengthening of villi affects digestion, improves absorption of nutrients, and promotes BWG. Therefore, alterations in intestinal morphology can affect nutrient absorption and animal performance. Polyphenols may exert effects on gut morphology. Feeding broilers with essential oils containing polyphenols caused an increase in villus height in the duodenal section of the gut [52]. This report was also supported by another study that concluded that hesperidin, genistein, and some flavonoids from Ginkgo biloba leaves increased absorptive surfaces of the small intestine by modifying its length, crypt depth, and villus width in broilers with lipopolysaccharide stress [53]. Chickens fed with diet supplemented with vitamin C and polyphenols presented an increase in length of the intestinal villi [54]. 

## 4. Class Specific Polyphenols-Gut Microbiota Interactions and Their Role in Modulation of Chicken Gut Microbiota 

The microbial transformations can differ depending on the type of polyphenolic structures (flavonoids or nonflavonoids), degree of polymerization and spatial configuration. Therefore, members of different subgroups and categories are metabolized through different pathways and result in different metabolic products causing differential effects on the gut microbiota (Table 2 and Table 3).

### 4.1. Flavonoids-Gut Microbiota Interactions and Their Role

Flavonoids (flavonols, flavanones, flavanols, isoflavones, flavones, and anthocyanins) have a backbone consisting of benzene rings (A and B) joined by pyrone C-ring [58]. Bacteria in the gut can cause cleavage of the C-ring from different positions, thus generating various simple phenolic moieties (Table 3). The position and pattern of B-ring hydroxylation influences the types of generated phenolic metabolites. Following C-ring-cleavage, flavonoids undergo further metabolism through demethylation and dehydroxylation reactions by the gut bacteria [59]. Different stains of *Lactobacilli* bacteria take part in the metabolism of flavonoids in the chicken gut [59].

Flavonoids and their microbiota derived metabolites show antimicrobial potential and have the ability to suppress pathogenic microbes [60]. Numerous *in vitro* studies showed that flavonoids from grape by-products can suppress certain bacteria such as *S. aureus*, *E. coli*, *C. albicans,* and *Campylobacter,* and can modulate composition of gut microbiota in chicken and improve their immunity and health [61,62]. Besides improving immunity, they affect gut functionality and promote gut morphology [61,62]. Genistein and hesperidin are known to regulate mucosal and cellular immunity [63]. Flavonoids [54] and flavonoids-rich fermented Ginkgo biloba leaves [64] have already been reported to promote intestinal morphology and absorption of nutrients in broilers. Hence, flavonoids can improve the gut morphometric index through the modulation of gut microbiota, providing protection against oxidative stress to epithelial cells [65]. However, the exact mechanisms by which flavonoids affect the intestinal architecture are yet to be described.

#### 4.1.1. Flavonols

Flavonols (kaempferol, quercetin, rutin, andmyricetin) are the flavonoids that have a 3–hydroxyflavone framework. These flavonoids are broken down by gut bacteria to produce simpler metabolites after C-ring cleavage. Gut microbes involved in these transformations include *Bacteroides distasonis*, *Bacteroides ovatus*, *Eubacterium ramulus*, *Enterococcus casseliflavus,* and *Bacteroides uniformis* [66]. Quercetin is the most studied member of this subgroup that is degraded by various bacteria including *Lactobacilli* [59]. 

Flavonols exhibit antimicrobial activities and can inhibit the growth of selective bacterial species. Among the flavonols, quercetin was reported to cause effective inhibition of bacteria like *Pseudomonas aeruginosa,* *Salmonella enterica* serotype Typhimurium, *Staphylococcus aureus,* and *Escherichia coli in broilers* [67]. Quercetin supplementation can also enhance performance efficiency in birds because it has the ability to modulate the intestinal architecture of chicken [56]. Rutin supplementation can improve FCR and promote BWG in broilers due to its favorable effects on gut morphology and small intestine functionality [25]. However, an *in vitro* study involving quercetin, myricetin, galangin, kaempferol, and fisetin reported only weak antimicrobial action of these flavonols against *B. adolescentis,* except galangin that caused effective inhibition (30 to 70%) of *B. adolescentis* [68].

#### 4.1.2. Flavanones

Flavanones (hesperetin, naringenin) are those flavonoids that share a 2,3-dihydro-2-phenylchromen-4-one backbone. These flavonoids appear to be more bioavailable as they are resistant to degradation by some colonic microbes. Deglycosylation and further metabolism of flavanones by gut bacteria appear to be similar to that of flavonols. Bacterial enzymes release the aglycones from flavanones like hesperidin and narirutin in colon. These aglycones are further transformed into phenolic acid moieties. Gut microbes such as *E. ramulus* and *Clostridium* species are capable of carrying out such colonic transformations [66]. Hydroxylated forms of phenylpropionic acid including 3-(3-hydroxy-4- methoxyphenyl)-propionic acid (dihydroisoferral acid) are noticed as colonic metabolites of hesperidin [73].

Prebiotic-like effects of hesperidin have been reported in the gastrointestinal tract of poultry [53]. Supplementation of feed with hesperidin (20 mg/kg diet) could improve gut health and boost immunity in poultry [53]. Glycosylated hesperidin also showed antibacterial action against pathogens like *Aeromonas hydrophila*. Sulphonated hesperidin, one of the plasma metabolites of hesperidin, can inhibit *Neisseria gonorrhoeae* and *Chlamydia trachomatis* [42]. Hesperetin aglycon (a citrus flavanone) can also inhibit *Helicobacter pylori* and vancomycin-intermediate *Staphylococcus aureus* (VISA) [74,75]. Naringenin favored the population of *Lactobacillus rhamnosus* as well as some disease-causing bacteria including *Escherichia coli*, *Staphylococcus aureus,* and *Salmonella thypi* [76]. The flavanones naringin and hesperidin also improved the FCR in broilers and enhanced the quality and oxidative stability of meat [77,78]. 

#### 4.1.3. Isoflavones

Isoflavones have planar basic ring system and have estrogenic properties. Almost all isoflavones are found in the glucosidal form and have very low absorption due to their high molecular weight and polarity. Their bioavailability depends on conversion of the glucosides to bioactive aglycones in the small intestine by enzymatic action of gut bacteria. Isoflavones are transformed into equol that is recognized for its positive health effects as compared to the primary isoflavones [79]. A study reported the conversion of isoflavone daidzein to equol in laying hens [69]. Gut microbiota of broilers can transform isoflavone glucosides into aglycones [57]. However, the metabolic pathway and bacteria involved in the biotransformation of isoflavones in poultry gut are yet to be described [69]. 

Isoflavones do affect the microbiota composition as some members of this subclass like daidzein and genistein have been reported to cause inhibition of certain gut bacteria [80]. They showed antimicrobial action against multiple resistant *S. aureus* (MRSA) strains [80]. Mechanism of their antimicrobial action could be due to the inhibition of bacterial topoisomerase IV [81]. Isoflavones can also favor certain bacteria in the chicken gut. Feeding of isoflavones-rich soybean meal increased the population of *L. delbrueckii*, *L intermedius*, *L. johnsonii*, *L. panis*, and *L. reuteri* in broilers [57].

#### 4.1.4. Flavanols

Flavanols comprise a complex subgroup of flavonoids containing simple flavanols (catechins, epicatechins, gallocatechins, epigallocatechins, and their gallate esters) and the corresponding polymeric structures. Bioavailability of flavanols is determined by their degree of polymerization and galloylation. Compounds having polymerization degree >3 pass to the colon unabsorbed where they are broken down [71]. Colonic bacteria break down the gallate esters. Epicatechin gallate and epigallocatechin gallate are transformed into aglycones that are further metabolized to pyrogallol [71]. The aglycones undergo C-ring cleavage and produce diphenylpropan-2-diol that is transformed into 5-(3′,4′-dihydroxyphenyl)-*γ*-valerolactone. Further metabolism yields OH-phenylpropionic and hydroxybenzoic acid moieties [71]. Hence, bioavailability and biological actions of flavanols are affected by the gut microbiota [82]. *Clostridium* (*Clostridium coccoides*) and *Bifidobacterium* (*Bifidobacterium infantis*) are considered responsible for these transformations [82].

Flavanols affect the microbial composition and their catabolic activities in the gut as some flavanol metabolites were reported to favor *Lactobacillus*/*Enterococcus* while causing a decrease in the population of *C. histolyticum* [43]. Catechin and its metabolites can inhibit *E. coli*, *Clostridium difficile* and *Salmonella* [83] without affecting *Lactobacillus* spp. [43]. High doses of flavanols have been linked with increased populations of *Bifidobacterium* in the colon. Polyphenon G powder (tea-derived catechins formulation) significantly increased *Lactobacillus* while markedly suppressing *Enterobacteriaceae* in broilers [84]. Catechins and epigallocatechins from tea also promote bacterial adhesion of *Lactobacillus rhamnosus* that supports mucosal defense [85]. Epigallocatechin shows antibacterial action against *Staphylococcus* and inhibits biofilm formations [86]. Epigallocatechin can also kill other bacteria including *Streptococcus pyogenes* [87], *Bacillus* spp. and *Clostridium* spp. [88], *Salmonella typhi* [89], and entero-hemorrhagic *E. coli* [90].

#### 4.1.5. Anthocyanins

The anthocyanins, which include pelargonidin, cyanidin, and malvidin exhibit very low absorption as only a small fraction of these compounds is hydrolyzed and transformed to absorbable metabolites. Hence, large quantities of ingested anthocyanins enter the colon [91] and interact with colonic microbiota [92]. Acylated forms of anthocyanins are also catabolized by gut bacteria. Gut bacteria break down the glycosidic bonds in anthocyanidins and open up the heterocycles. Cleavage of 3-glycosidic linkage by gut bacteria was confirmed by Keppler and Humpf [92]. Bacteria involved in breakdown of anthocyanins are *Bifidobacterium lactis*, *Lactobacillus plantarum*, *Lactobacillus acidophilus,* and *Lactobacillus casei* [72]. Anthocyanins are first deglycosylated and then degraded into simpler phenolic acids by colonic microflora. For example, malvidin-3-glucoside is transformed into syringic, gallic, and p-coumaric acids by fecal bacteria. Protocatechuic acid is identified as the main gut microbiota derived metabolite of anthocyanidin. Other microbial derived metabolites of anthocyanins are phloroglucinol and vanillic [92,93]. 

Anthocyanins and their metabolites can regulate changes in the gastrointestinal tract by favoring the growth of beneficial bacteria like *Lactobacillus* and *Enterococcus* [51]. They can ameliorate heat stress in chickens and can serve as feed additives in poultry production. Feeding of anthocyanin-rich cranberry extract containing 40, 80, or 160 mg/kg anthocyanins resulted in superior FCR and improved BWG in broilers [44]. Anthocyanins also improve organ functionality and reduce pathogenic bacteria in the poultry gut. They favor *Bifidobacterium* spp., *Enterococcus* spp., and *Lactobacillus* spp., and can result in favorable microbiota modulation [51]. Anthocyanins can also suppress pathogenic bacteria. Birds fed with high concentrations of cranberry extract containing 160 mg/kg of anthocyanins decreased *Enterococcus* spp. population in caeca and cloaca [94]. 

#### 4.1.6. Flavones

Flavones (luteolin and apigenin) occur in the glycosylated form in nature. These flavonoids are transformed into aglycones by some *Lactobacillus* bacteria including *Lactobacillus agilis* in the chicken gut [59]. Once the glucosides are hydrolyzed in the intestines, the unabsorbed aglycones undergo further degradation through C-ring cleavage by colonic bacteria and are converted to absorbable metabolites [59]. 

Flavones and their metabolites can affect gut health in poultry. Alfalfa flavones enhanced growth efficiency in chickens [95]. Flavones have shown antibacterial potential against various microbes. Particularly, luteolin exhibited antibacterial potency against amoxicillin resistant *E. coli* [96]. It also suppressed *S. aureus*, *E. coli*, *B. subtilis,* and *P. fluorescens* [97]. Luteolin and its 4′-*O*-glucoside are bactericidal to *E. coli* at minimum inhibitory concentration of 1.0 × 10^−1^ mg/mL and *S. aureus* at 5.0 × 10^−2^ [98].

### 4.2. Non-Flavonoids-Gut Microbiota Interactions and Their Role

Non-flavonoids comprise a complex and heterogeneous group of polyphenols ranging from the simplest hydroxybenzoic acids and hydroxycinnamic acids to complex stilbenes, lignans, and tannins [37]. They vary in complexity of structure among different subclasses and show different levels of bioavailability and their metabolism in the gut varies depending on the level of complexity of their structures among different subclasses [37] (Table 4). 

#### 4.2.1. Phenolic Acids

Phenolic acids are the compounds having both carboxylic acid (-COOH) and hydroxyl (-OH) groups attached to the benzene ring. They can be divided into hydroxy benzoic acids and hydroxy cinnamic acids [37]. The general structure of hydroxy benzoic acids can be abbreviated as C_6_–C_z_ whereas hydroxycinnamic acids are the derivatives of cinnamic acids and belong to phenylpropanoids having a C_6_–C_3_ backbone [99]. Protocatechuic acid, vanillic acid, syringic acid, and *p*-hydroxybenzoic acid are the four predominant members of hydroxy benzoic acids, whereas caffeic acid, *p*-coumaric acid, ferulic acid, and sinapic acid belong to hydrocinnamic acids [37]. 

A general trend in the metabolism of phenolic acids is that the free acid is released by bacterial esterases and double bond is then reduced to produce phenylpropionic acid and further decarboxylated to yield phenylacetic acids. Reduction follows dehydroxylation resulting in the removal of hydroxyl at C4-position of hydroxycinnamic acid residue. Gallic acid (3,4,5-trihydroxybenzoic acid) generates pyrogallol (1,2,3-trihydroxyphenol), and protocatechuic acid (3,4-dihydroxybenzoic) is converted to catechol (1,2-dihydroxyphenol). Similarly, vanillic acid (3-methoxy-4-hydroxybenzoic acid) is transformed into *O*-methylcatechol [100]. Caffeic acid produces 3-hydroxyphenylpropionic acid and benzoic acid. Both these metabolites are also generated from chlorogenic and caftaric acid, suggesting that esterification does not affect metabolism of caffeic acid by gut microbiota. *Bifidobacterium* and *Lactobacillus* seem to be involved in transformation of chlorogenic acid [101]. Microbiota derived metabolites of ferulic acid include vanillin and 3-(4-hydroxyphenyl)-propionic acid. In vivo phase II metabolism of microbial metabolites of caffeic acid generates glycynated metabolites that includes hippuric acid and 3-hydroxyhippuric acid [66].

Phenolic acids and their microbial metabolites affect intestinal bacteria composition and their metabolic activity [102]. 3-*O*-methylgallic acid, caffeic acid, and gallic acid can decrease the population of pathogens (*C. perfrigens*, *C. difficile,* and *Bacteroides* spp.) without affecting commensal anaerobic bacteria (*Bifidobacterium* spp.) and probiotic bacteria (*Lactobacillus* spp.) [103]. Gallic acid prevents the formation of biofilms as it reduced biofilm formation activity of *E. coli*, *P. aeruginosa*, *S. aureus,* and *L. monocytogenes* by >70% [104]. It further inhibits the growth of *Streptococcus mutans* and affects the adhesion properties of *S. aureus* [104]. Furthermore, gallic acid supplementation can affect the morphology of the gut and enhance the absorption of nutrients by improving the villus height to crypt depth ratio in broilers [41].

Hydroxycinnamic acids like chlorogenic acid, *p*-coumaric acid, coumaric acid, and caffeic acid affected *Escherichia coli*, *Bacillus cereus,* and *Staphylococcus aureus* [105]. Caffeic acid has been reported as the strongest inhibitor of the growth of *E. coli*, *Salmonella*, *Pseudomonas*, *Clostridium,* and *Bacteroides* [103]. 

#### 4.2.2. Tannins

Despite their complex structure, tannins are metabolized by gut microbiota. Gut bacteria have the ability to hydrolyze ester bonds in tannins through tannin acyl hydrolase activity [109]. Hydrolysis of tannin-*O*-glycosides produces gallic acid residues from gallotannins and ellagic acid from ellagitannins. Ellagic acid dehydroxylation produces nasutins in which two hydroxyl groups are removed [109]. Ellagic acid degradation also takes place by lactone-ring cleavage and decarboxylation. Ellagic acid is transformed into urolithins in the mammalian gut. However, the transformation of ellagic acid into urolithins in the chicken gut has not been reported [110]. 

Tannins caused an increase in *Bacteroidetes* and *Firmicutes* in caecal microbial communities of chickens [111]. Tannins can induce iron-poor conditions and support the growth of *Lactobacillus* bacteria, as these bacteria do not require iron for their growth [112]. Dietary tannin supplementation has also been associated with a reduction in the population of *Bacteroides* resulting in decreased acetate and propionate production. However, increased population of *Clostridiales,* specifically the members of *Ruminococcaceae,* compensates for this decrease by producing butyrate [113]. An overall rise in short chain fatty acid production in poultry has been associated with tannic acid supplementation [114]. With regards to morphology, sorghum tannins did not show any adverse effects on intestinal morphology in chickens or laying hens [115]. However, faba bean tannins have been reported to cause atrophy and shortening of villi in chicken [116].

#### 4.2.3. Lignans

Lignans are polyphenols with a 1,4-diarylbutane structure and contain diphenolic compounds. During metabolism, plant lignans undergo glucuronidation and to a lesser extent sulphation [117]. Biological actions of lignans depend on their conversion to their phytoestrogenic forms, i.e., enterolactone and enterodiol [108]. These enterolignans are produced by gut microbiota. The presence of microorganisms capable of converting lignans into enterolignans is important for their health benefits [117]. Lignans metabolism by gut microbiota involves demethylation, reduction, dehydroxylation, and lactonization [108], and phytoestrogens are produced by *Eubacterium* and *Peptostreptococcus* bacteria. Pinoresinol and lariciresinol undergo benzyl ether reduction, guaiacol demethylation, catechol dihydroxylation, and diol lactonization reactions and are transformed via various intermediary compounds into enterodiol and enterolactone from dietary lignans [118]. However, it is difficult to predict a definite relation between lignans and gut microbiota due to lack of scientific reports in this area. 

#### 4.2.4. Stilbenes

Stilbenes have a C6-C2-C6 structure and can be metabolized by gut bacteria. Trans-resveratrol is the most studied stilbenoid due to its well-known health-effects [119]. Gut microbes are able of metabolize resveratrol. Resveratrol and its precursors including piceid undergo microbial metabolism in the gut and can produce bioactive metabolites like dihydroresveratrol. Little is known about the fate of resveratrol in the chicken gut due to scarcity of scientific literature. Resveratrol has very low bioavailability [13]. Therefore, resveratrol can play a role in modulation of the gut microbiota of chicken due to its antimicrobial potential. Resveratrol exhibits antibacterial activity against *Salmonella enterica*, *Enterococcus faecalis,* and *Escherichia coli* [120]. Resveratrol also suppresses the growth of *E. coli* while causing a significant increase in *Lactobacilli* and *bifidobacteria* [38]. Another study supported these results and reported an increase in *Lactobacilli* and *bifidobacteria* while decreasing *Clostridium perfringens* in the chicken gut [121]. Resveratrol also improved villus height to crypt depth ratio by increasing villus height and lowering the crypt depth [38]. Hence, resveratrol can improve immunity, gut health, and growth performance by affecting gut permeability and composition of microbiota in chickens [122]. 

## 5. Conclusions

A healthy gut system is of prime importance in poultry production. Detrimental changes induce dysbiosis in the gut, which results in poor performance and a decline in overall health of chickens. Polyphenols supplementation can improve gut health and productivity of the chicken. Health promoting properties of these phenolic compounds are attributed to their effects on gut microbiota. The gut microbiota-polyphenol interactions are a two-way process in which gut microbes transform polyphenols into their bioactive metabolites having improved bioavailability and health effects, whereas polyphenols and their gut microbiota derived metabolites can support growth of beneficial bacteria and inhibit pathogens. Study of gut microbiota-polyphenols interactions is an emerging field of interest due to its importance in gut health and modulatory effects. Further research should, therefore, be focused on understanding gut microbiota-polyphenols interaction, their effects on gut microbiota, meat quality, and overall health of poultry by conducting feeding trials using different plant sources containing polyphenols to develop an optimum feeding strategy for sustainable poultry production.

## Figures and Tables

**Table 1:** Correlation of microbial taxa with productive performance of chicken.

Sample	Performance Parameter	Microbial Taxa Identified		References
		Higher Productive Performance	Lower Productive Performance	
Crop	BW	*Faecalibacterium*, *Euryarchaeota*, *Bacteroidetes*, *Ruminococcus*	*Bifidobacterium*, *Actinobacteria*, *Lactobacillus*	[17]
	BW	*C. coccoides*	*Shigella*, *E. coli*, *Enterobacteria*	[18]
Duodenum	RFI	*Lactobacilli*	*Bacteroides*	[21]
Jejunum	FCR	No difference	No difference	[15]
	FCR	*Lactobacillus*, *Fructobacillus*, *Paralactobacillus*	*Leptotrichia*, *Pediococcus*, *Rohdococcus*, *Escherichia coli*	[16]
Ileum	BW	*Spirochaetes*, *Euryarchaeota*, *Bifidobacterium*, *Methanobrevibacter*	*Akkermansia*, *Streptococcus*	[17]
	BW	*Bacteroides*	*-*	[18]
	RFI, TFI, TBWG	*Enterobacteriaceae*	*Turicibacter*, *Lactobacillus and Ruminococcus*	[20]
	FCR	*Enterococcus*, *Clostridium*, *Pseudanabaena*, *Bacillus*, *Mannheimia*, *Granulicatella*	*Halochromatium*	[16]
Ileum—Cecum	RFI	*Turicibacter*, *Coprococcus* and *Ruminococcus,*	*Proteobacteria* and *Clostridiales*	[30]
	BW	*Lactobacillus*, *Actinobacteria*, *Firmicutes* and *Tenericutes*	*Clostridium*, *Firmicutes*, *Bacteroidetes* and *Proteobacteria*	[31]
	BW	*Butyricimonas*, *Bilophila*, *Faecalibacterium* and *Bacteroides*	*Enterococcus*, *Coprococcus*, *Anaerotruncus*, *Coprobacillus*, *Bacteroides*, *Clostridium*, *Streptococcus*, *Lactobacillus*, *Ruminococcus*, *Staphylococcus* and *Enterobacteriaceae*	[32]
Cecum	FCR	*B. fragilis*	*Ruminococcus*, *Clostridiales* and *L. crispatus*	[15]
	RFI	*Prochlorococcus marinus*, *Akkermansia*, *L. reuteri*, *L. delbrueckii*, *Prevotella*, *B. coprophilus* and *L. Veillonella dispar*	*Parabacteroides distasonis*, *Helicobacter*, *F. prausnitzii*, *Thermobispora bispora*	[21]
	FCR	*R. torques*, *F. prausnitzii* and *C. lactatifermentans*	*B. vulgatus* and *Alistipes finegoldii*	[33]
	BW	*Lactobacillus*	*Escherichia*, *Campylobacter* and *Shigella*	[14,18]
Feces	RFI	*Helicobacter*	*Lactobacillus* and *Clostridium*	[21]
	TBWG, RFI, TFI	*Enterobacteriaceae* and *Lactobacillaceae*	*Acinetobacter*, *Comamonadaceae* and *Moraxellaceae,*	[34]
	FCR	*Synergistaceae*, *Victivallaceae*, *Prevotellaceae*, *Rikenellaceae*, *Enterobacteriaceae* and *Ruminococcaceae*	*Xanthomonadaceae*, *Incertae*, *Vibrionaceae*, *Fusobacteriaceae*, *Campylobacteraceae*, *Rhizobiaceae*, *Flavobacteriaceae, Comamonadaceae*	[22]
	TBWG, RFI, TFI	*L. salivarius*, *Anaerobacterium*, *L. crispatus*	*Klebsiella*	[35]

AME: apparent metabolizable energy; FCR: feed conversion ratio; BW: body weight; RFI: residual feed intake; TFI: total feed intake; TBWG: total body weight gain.

**Table 2:** Gut Microbiota-polyphenols interaction and its effects on the gut.

Polyphenol	Bacteria Involved	Mechanism	Effect	References
Curcumin	*Firmicutes*, *Enterobacteriaceae*	Antibacterial activity	Improved antioxidant potential/Modulation of gut microbiota	[55]
Naringenin/Hesperetin	*Escherichia coli*, *Helicobacter pylori*, *Salmonella aureus*	Antibacterial activity	Affected upper part of gut, altered gut bacteria patterns	[41]
Gallic acid		Inhibitory effect on the growth of jejunum villi	Reduced crypt depth and increased villus height to crypt depth ratio	[41]
Quercetin	*Bifidobacteria* and coliforms	Antibacterial activity	Modulation of cecal bacteria population	[56]
Soybean polyphenols	*L. delbrueckii*, *L intermedius*, *L. johnsonii*, *L. panis*, *L. reuteri*	Prebiotic effect	Modulation of gut microbiota	[57]
Grape polyphenols	*Escherichia coli* and lactic acid bacteria	Antimicrobial action	Reduced *Escherichia coli* and lactic acid bacteria in the ileum	[29]
Blueberry polyphenols	*Lactobacillus, Bacteroides, Parabacteroides, Bifidobacterium*	Prebiotic effect	Modulation of gut microbiota	[48]
Resveratrol	*E. coli*	Antibacterial activity	Ameliorated dysbiosis caused by heat stress	[38]

**Table 3:** Main gut microbiota transformed flavonoid metabolites and the bacteria involved.

Subclass	Compounds	Metabolites Identified	Bacteria Involved	References
Flavonols	Rutin	Quercetin	*Lactobacilli*	[59]
	Quercetin		*Lactobacilli*	[59]
Flavanones	Hesperidin	Hesperitin	*Lactobacilli*	[59]
Isoflavones	Daidzein	Equol		[69]
		*O*-demethylangolensin		[70]
	Genistein	6′-hydroxy-*O*-desmethylangolensin		
Flavanols	Catechin	3-(3-hydroxyphenyl)propionic acid		
	Epicatechin	3-(3,4-dihydroxyphenyl)propionic acid		
		5-(3,4-dihydroxyphenyl)valeric acid, 5-(3′,4′-dihydroxyphenyl)-*γ*-valerolactone	*Bifidobacterium* spp., *Clostridium coccoides*	[71]
		5-(3′,5′-dihydroxyphenyl)-*γ*-valerolactone		
	Epigallocatechin	5-(3′,4′-dihydroxyphenyl)-*γ*-valerolactone		
Anthocyanins	Peonidin	3-methoxy4-hydroxybenzoic acid	*Lactobacillus plantarum*, *Bifidobacterium lactis* BB-12, *Lactobacillus acidophilus* LA-5, *Lactobacillus casei*	[72]
	Malvidin Pelargonidin	3,4-dimethoxybenzoic acid		
	Cyanidin	4-hydroxybenzoic acid		
		3,4-dihydroxybenzoic acid		

**Table 4:** Gut microbiota derived nonflavonoids metabolites and bacteria involved.

Subclass	Compounds	Metabolites Identified	Bacteria Involved	References
**Phenolic acids**	Caffeic acid, Ferulic acid	Hydroxyphenyl-ethanol 3-(4-hydroxyphenyl)-propionic acid 3-(4-hydroxyphenyl)-propionic acid Phenylacetic acid 3-(3-hydroxyphenyl)-propionic acid 3-(3,4-dihydroxyphenyl)-propionic acid Benzoic acid	*Lactobacillus gasseri*, *Bifidobacterium lactis*, *Escherichia coli*	[106]
**Tannins**			*Lactobacillus, Enterococcus*	[107]
**Lignans**		Enterodiol Enterolactone		[108]
